# Meaningful objects avoid attribute amnesia due to incidental long-term memories

**DOI:** 10.1038/s41598-023-41642-z

**Published:** 2023-09-02

**Authors:** Edyta Sasin, Yuri Markov, Daryl Fougnie

**Affiliations:** 1 Department of Psychology, New York University Abu Dhabi, Abu Dhabi, UAE; 2 Department of Psychology, Goethe University Frankfurt, Frankfurt am Main, Germany

**Keywords:** Psychology, Human behaviour

## Abstract

Attribute amnesia describes the failure to unexpectedly report the attribute of an attended stimulus, likely reflecting a lack of working memory consolidation. Previous studies have shown that unique meaningful objects are immune to attribute amnesia. However, these studies used highly dissimilar foils to test memory, raising the possibility that good performance at the surprise test was based on an imprecise (gist-like) form of long-term memory. In Experiment 1, we explored whether a more sensitive memory test would reveal attribute amnesia in meaningful objects. We used a four-alternative-forced-choice test with foils having mis-matched exemplar (e.g., apple pie/pumpkin pie) and/or state (e.g., cut/full) information. Errors indicated intact exemplar, but not state information. Thus, meaningful objects are vulnerable to attribute amnesia under the right conditions. In Experiments 2A-2D, we manipulated the familiarity signals of test items by introducing a *critical object* as a pre-surprise target. In the surprise trial, this critical item matched one of the foil choices. Participants selected the critical object more often than other items. By demonstrating that familiarity influences responses in this paradigm, we suggest that meaningful objects are not immune to attribute amnesia but instead side-step the effects of attribute amnesia.

Our visual world is complex, yet we effortlessly recognize countless objects every day. Perhaps even more impressive is how much of this complex world we can encode and remember later. Studies on visual long-term memory (LTM) show good recognition memory for the brief visual presentation of thousands of objects^1–3^. However, other evidence suggests that memory can be quite limited. While we intuitively believe that we represent most of our visual world, several studies reveal that we encode very little^4–7^. For example, studies on *attribute amnesia* (AA) reveal that people can fully process a feature of an object, only to fail to report it a moment later. For example, one study^8^ had participants report the location of a letter (among digits) during multiple trials. On a surprise trial, participants were unable to report the identity of the letter. However, their performance dramatically improved when they were asked the same question on the post-surprise control trials. The authors proposed that AA is likely due to a lack of memory consolidation. That is, even if a particular attribute is encoded when the task is executed, it is not consolidated in working memory into a robust memory trace.

Are there limits on the types of attributes that people can encode but not consolidate in working memory? The AA effect has largely been studied (and replicated) across a range of simple stimuli such as colors, letters, and digits^9–12^. However, what about complex, meaningful objects? Such stimuli show impressive recall in the context of long-term memory studies. Further, there is a clear advantage when processing and remembering complex, meaningful objects relative to simple objects^13–16^. These facts have led researchers to propose that meaningful objects are a special class of stimuli that are immune to the factors that produce AA^17^. Consistent with this, existing work largely shows that unique meaningful stimuli are not susceptible to AA^17,18^, with only one study finding any effect for such stimuli^19^.

But what is it about meaningful stimuli that allow them to avoid AA? Importantly, AA is thought to reflect a failure to consolidate an attended feature into an active working memory representation^20^. One possibility is that meaningful stimuli are more successfully supported by memory systems such as long-term memory (LTM)^21–23^, which are not vulnerable to AA. Thus, meaningful stimuli may be susceptible to the mechanisms that produce AA, but successful surprise trial performance for meaningful stimuli may be from information in LTM rather than from an active working memory representation.

Two attributes of AA studies with meaningful stimuli raise the possibility that LTM could be particularly effective in these studies. First, previous studies using meaningful stimuli found AA to be absent when objects were unique, i.e., stimuli were not repeated during the experiment. However, AA reappeared when meaningful stimuli were repeated across trials^17,18^. These findings are suggestive that the benefit from meaningful objects in past studies could arise due to a contribution from LTM, particularly from familiarity signals (a general sense that an object is familiar based on previous exposure to it). Familiarity signals would be effective only when stimuli are not repeated because they are inefficient for determining whether an item was seen on the current or previous trial. Thus, stimulus repetition leads to conditions in which an active memory is required. Importantly, while one study^17^ did find AA for simple stimuli in the absence of stimulus repetition, the simple stimuli used were all already highly familiar (e.g., letters and numbers) and also more similar to each other than the meaningful stimuli sets in other AA experiments^17,18^.

Second, previous studies require differentiating between visually and semantically dissimilar objects (e.g., target bed and non-targets: table, couch, lamp). Thus, knowing only the semantic category or basic meaning (e.g., you remember seeing some car, and not a truck, but without remembering the type of car or the color of the car) would be sufficient for good performance. Critically, such a memory representation may not require memory of any visual details. Further, by having semantically dissimilar correct and foil probes, the heterogeneity of stimuli will produce strong familiarity signals similar to the benefit of high target-distractor dissimilarity in visual search^24^. Thus, even an imprecise LTM representation containing basic meaning could support performance.

Broadly, an issue with previous studies that examined AA with meaningful stimuli is that these studies used memory tests with low sensitivity. Having a limited, incomplete memory is sufficient for performance, such as a gist-level representation without any visual details. One goal of the present work was to examine whether a more sensitive test of memory would reveal an AA for memory of unique meaningful stimuli. We adopted a general AA framework in which we presented a display of four real-world objects from different categories and asked participants to localize an item from target category (food object). After numerous trials, we presented a surprise trial that had participants select the recently attended object from a set of possible choices. Critically, we utilized a more sensitive four-alternative forced-choice (4-AFC) surprise test consisting of the correct item (e.g., full apple pie), an item with a matching state but mis-matched exemplar (e.g., a full pumpkin pie), an item with a matching exemplar but mis-matched state (e.g., a cut apple pie), or an item with mis-matching exemplar and state (e.g., a cut pumpkin pie). From this, we can separately analyze state and exemplar errors in the surprise (and control) trials. No previous study has examined whether an AA for meaningful objects would be found when the task requires a more sensitive comparison (such as an exemplar and state judgment). Previous studies used semantically and visually different foils (e.g., different types of furniture or animals) on the surprise trial. Such a comparison could be successfully executed only based on semantic-gist representation (e.g., “it was a cat and not a dog”). On the contrary, distinguishing between different exemplars and states (e.g., different breeds of cats in a different position) cannot be based only on general semantic-gist representations but requires specific details (e.g., “it was a Persian cat standing”).

Further, a second goal was to induce manipulations of LTM signals for items during the surprise trial to test how these signals can modulate the strength of AA. To do this, we conducted Experiments 2A-2D, in which, similar to Experiment 1, people were searching for a food object on multiple trials before being unexpectedly asked to identify the target object in the 4 AFC test. Crucially, on one of the pre-surprise trials, we presented a *critical object*: a target object that was mis-matched in exemplar or state or both attributes, or it was the same as the target object in the later surprise trial. We predict that performance on the surprise trial would depend on which critical object was processed on the earlier trial. Specifically, we expect reduced AA for trials in which the critical object constituted the correct choice in a surprise test (Experiment 2D) and increased exemplar and state errors when the critical object mis-matched in the respective attribute. Notably, the manipulation of LTM signals in the current study is more sensitive than in previous experiments that either repeated objects or not. In contrast to previous studies, here, we manipulated the familiarity of particular target attributes, and we presented a target-related stimulus only once.

## Experiment 1

### Method

#### Participants

Forty observers (21 females, mean age = 24.73 years, SD = 5.63) were recruited and run via an online platform, Prolific (www.prolific.ac^25,26^). We doubled the sample size of 20 participants used by previous studies with similar paradigm^17,18^, expecting more noise due to online data collection. All participants gave informed consent and were compensated 0.6£ for approximately 7 min of their time. The experiment was approved by the New York University Abu Dhabi Institutional Review Board in accordance with the ethical principles of the Belmont Report.

### Apparatus and stimuli

The experiment was created and controlled using PsychoPy v2020.2.10^27,28^ and hosted online on www.pavlovia.org website. All stimuli were displayed on a white background. A black fixation cross (20 × 20 pixels) was presented centrally throughout the whole experiment (except the surprise test screen). At the start of each trial, four black placeholder circles (diameter 50 pixels, line width 4 pixels) were displayed equidistantly on an invisible circle (radius 170 pixels) around fixation. A search display consisted of three distractor images and one target image presented at the same locations as placeholders. The mask was composed of four squares (each 200 × 200 pixels) filled with randomly colored pixels (for mask generation, the following code was used https://github.com/Robson/White-Noise-Image-Generator). Images used in the experiment were obtained from various resources, including Google Images, 123RF image database (www.123rf.com), images pulled from Brady et al.^29^ image set, and Markov, Utochkin and Brady^30^ image set. Each image in the search display was selected from a different category and fit a square of 180 × 180 pixels. The distractors were drawn from animals, clothes, and transportation categories, and the targets were drawn from a food category. Each category set included two exemplars of each object (e.g., two different exemplars of a cat or two different exemplars of a bicycle). The images in the search task were selected at random with the restriction that only one image from the exemplar pair was presented in the experiment, and no image was repeated across trials. There were 54 exemplar pairs of distinct animals, 59 exemplar pairs of distinct clothes, and 56 exemplar pairs of distinct transportation vehicles. The 48 distinct exemplars pairs of food objects were used only for pre-surprise trials. For surprise and control trials, additional target images were selected consisting of 21 exemplar pairs of food objects occurring in two different states (21 × 2 exemplars × 2 states), e.g., two different peanut butter jars in two states: open or closed.

**Figure 1:**
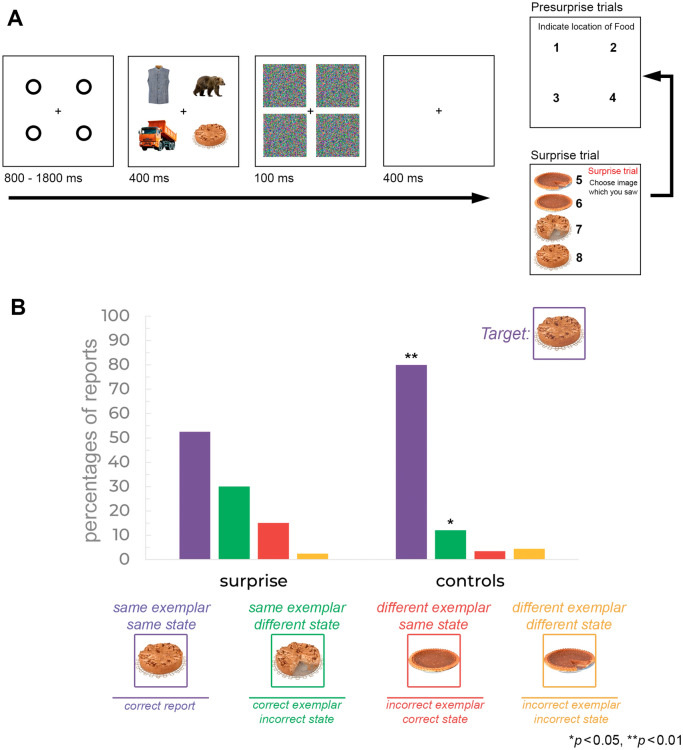
(**A**) Example of trial sequence in Experiment 1. For the 30 pre-surprise trials, participants localized the food object. On the 31st (surprise) trial, participants were to indicate which target object had been presented by selecting one of four alternatives. The localization task followed the surprise question. After the surprise trial, participants executed five control trials, including the target identification task and localization task, as in the surprise trial. (**B**) Percentage of different reports on surprise and control trials. Percentages of reports in pre-surprise and control trials constitute the mean across trials and participants. Stars indicate a significant difference between surprise and control trials tested by chi-square.

There were three types of trials in the experiment. On *pre-surprise* trials, participants were asked to localize the target (food) object. On the *surprise* trial, an unexpected identification test was presented, requiring participants to select the target object that they had just seen in this trial. Four alternatives were shown (see Fig. 1A): the *same* exemplar in the *same* state (target object, e.g., full apple pie), the *same* exemplar in a *different* state (sliced apple pie), a *different* exemplar in the *same* state (full pumpkin pie) and a *different* exemplar in a *different* state (sliced pumpkin pie). The position of the four alternatives was randomized. After the unexpected identification test, participants had to report the location at which the target object was presented. Following the surprise trial, the five *control* trials with a new set of stimuli were presented. These control trials were identical in procedure to the surprise trial.

### Procedure

An example of the stimuli and procedure is shown in Fig. 1A. Each trial started with the presentation of four placeholder circles for 800 ms to 1800 ms. The timing was varied to be unpredictable for participants. Participants then viewed a search display of four images for 400 ms, followed by pattern dot masks for 100 ms, and followed by a 400-ms blank interval. For the first 30 *pre-surprise* trials, four numbers (from 1 to 4) then appeared at the locations of four images. Participants pressed one of the number keys to indicate the location of the target object. On the 31st trial (*surprise* trial), observers were unexpectedly asked to identify the target object by selecting one of four alternative images presented vertically on the screen. Participants responded by pressing one of four number keys (5–8) corresponding to the numbers displayed next to the images. Once a response was made, observers were asked to report the location of the target in the same way as in the *pre-surprise* trials. Following the 31st trial, the observers executed five *control* trials identical to the *surprise* trial.

## Results

To compare performance in different types of trials, we performed chi-square tests (chisq.test, RStudio Team^31^) on frequencies of each type of reports in the surprise test and the mean frequencies in pre-surprise and control trials.

### Localization task

The errors in the target localization task in pre-surprise trials were low (4.42%), indicating that participants could easily find the target food image among distractor images from different categories. We observed a drop in performance in the location task on surprise compared to pre-surprise trials (4.42% vs. 32.5% pre-surprise vs. surprise trials, respectively, *χ*2[1, *N* = 40] = 10.48, *p* = 0.001207, *ϕ* = 0.51). This drop was presumably due to additional demands imposed on WM by the unexpected question, resulting in a higher likelihood of forgetting the location. However, once participants became familiar with the new procedure, their performance on the location task improved, which is reflected in better performance on control than surprise trials (32.5% vs 12.5%, surprise vs. control trials, respectively, *χ*2[1, *N* = 40] = 4.5878, *p* = 0.0322, *ϕ* = 0.0339). A similar decrease in location task performance on surprise trials and improvement on control trials has been observed in previous experiments^17^.

### Identification task

Rather than simply looking at performance accuracy, we break down responses into the four possible choices in the identification task. Specifically, participants could select the correct target object: the *same* exemplar in the *same* state or make one of three types of errors. They could select the *same* exemplar in a *different* state, a *different* exemplar in the *same* state, or a *different* exemplar in a *different* state. Percentages of the *same* exemplar in the *same* state reports were significantly lower in surprise trials (52.5%) compared to control trials (80%, comparison: *χ*2[1, *N* = 40] = 6.7645, *p* = 0.0093, *ϕ* = 0.41, Fig. 1B). We found significantly more the *same* exemplar in a *different* state reports in surprise trials (30%) compared to control trials (12%, comparison: *χ*2[1, *N* = 40] = 3.906, *p* = 0.0481, *ϕ* = 0.31, Fig. 1B). There was no difference between surprise and control trials in a *different* exemplar in the *same* state reports (15 vs. 3.5%, *χ*2[1, *N* = 40] = 3.1509, *p* = 0.075, *ϕ* = 0.28, or a *different* exemplar in a *different* state reports (2.5 vs. 4.5%, *χ*2[1, *N* = 40] = 0.23686, *p* = 0.6265, *ϕ* = 0.08).

These data show that meaningful objects can be shown to be vulnerable to AA with a sensitive memory probe. The state judgment errors were higher in surprise than in control trials. However, the data also suggest that the memory of the target object was generally sufficient to distinguish between two exemplars in the surprise test, given that exemplar errors (Fig. 1) were low, and these errors did not differ between surprise and control trials. From these findings, we suggest that the representations of meaningful objects that survive AA are more than gist-like representations. They include some features allowing for exemplar distinction, but at the same time, they are not detailed enough, or the memory signal is not strong enough, to allow for good performance in state judgments.

While this experiment does not directly test the question of whether successful avoidance of AA for meaningful objects is due to LTM, it does place important limits on the conditions upon which AA can be avoided. Further, these limits are highly suggestive of a role for LTM given that we found a significant difference in state errors between surprise and control trials. Suppose judgments on the surprise trial were primarily based on working memory rather than LTM. In that case, we should not observe AA for only state errors, as working memory engagement should also prevent higher errors for state information.

To provide a more direct of the influence of LTM in the AA task, we conducted Experiments 2A-2D. In these experiments, we manipulated memory signals of test items by increasing familiarity. Specifically, one of the four items during the surprise target identification task was shown early in the experiment as a target —thus, it had to be categorized and localized as a food object. Of interest was whether processing the critical object at the category level earlier in the experiment would lead to it being chosen at a higher frequency on surprise trials. This manipulation of familiarity (the repeated item having higher familiarity) would provide more direct evidence of a role in familiarity/LTM in the surprise task and thus provide supporting evidence that the ability of meaningful objects to avoid AA may be due to contributions from LTM. Because there were four distinct response choices, we conducted four versions of the study. In Experiment 2A, the critical object (compared to the target from a surprise trial) was the *same* exemplar in a *different* state. In Experiment 2B, it was a *different* exemplar in the *same* state. In Experiment 2B, it was a *different* exemplar in a *different* state. In Experiment 2D, it was the correct response—the *same* exemplar in the *same* state.

## Experiments 2A-2D

### Method

#### Participants

As in Experiment1, forty observers were recruited in each experiment (16 females, mean age = 23.6 years, SD = 4.9 in Experiment 2A; 10 females, mean age = 25.1 years, SD = 5.6 in Experiment 2B;10 females, mean age = 23.4 years, SD = 4.44 in Experiment 2C and 22 females, mean age = 23.9 years, SD = 4.8 in Experiment 2D). Participants were again recruited via Prolific (www.prolific.ac^25,26^. All participants gave informed consent and were compensated 0.6£ for approximately 7 min of their time. The experiment was approved by the New York University Abu Dhabi Institutional Review Board in accordance with the ethical principles of the Belmont Report.

### Apparatus, stimuli, and procedure

Apparatus, stimuli, and procedure were identical to Experiment 1, except that we presented six different *critical objects* as targets in localization task during *pre-surprise* trials (chosen at random from the 3rd to 28th trial) that were then repeated as one of the response choices in surprise and the five control trials. Each of the six critical objects was repeated once and its relationship with the target when repeated was manipulated across studies. They could either be the *same* exemplar in a *different* state (Experiment 2A), a *different* exemplar in the *same* state (Experiment 2B), a *different* exemplar in a *different* state (Experiment 2C), or the *same* exemplar in the *same* state (Experiment 2D). Importantly, while surprise and control trials involved different critical objects, the object’s relationship to the target was identical between these trial types within each experiment.

## Results

We hypothesized that participants would select the critical item more frequently as a result of its increased familiarity. There were four possible relations between the critical object and the target response, which were each contained in a study (e.g., the critical object was the correct answer in 2D but was the wrong exemplar and state in 2C). Pooling across studies makes each response choice equally likely to have been a critical object. Therefore, we can examine whether the *critical object* was selected at a higher-than-chance rate in the pooled data. To illustrate that chance performance is expected if the critical object is not exerting an influence, regardless of the participant’s ability to identify the target, we can consider the edge cases. If participants have no information about the target, responses will be random relative to the critical object, and the expected chance that the critical object will be selected for each version of the study will be 25%. However, if participants always know the target, the proportion of responses that match the critical object will still be 25% overall (100% for 2D and 0% for A-C). Therefore, pooling is useful in eliminating baseline differences in different response choices (since all response choices are equally represented) and is practical given the low power caused by only having one surprise trial/response per participant.

**Figure 2:**
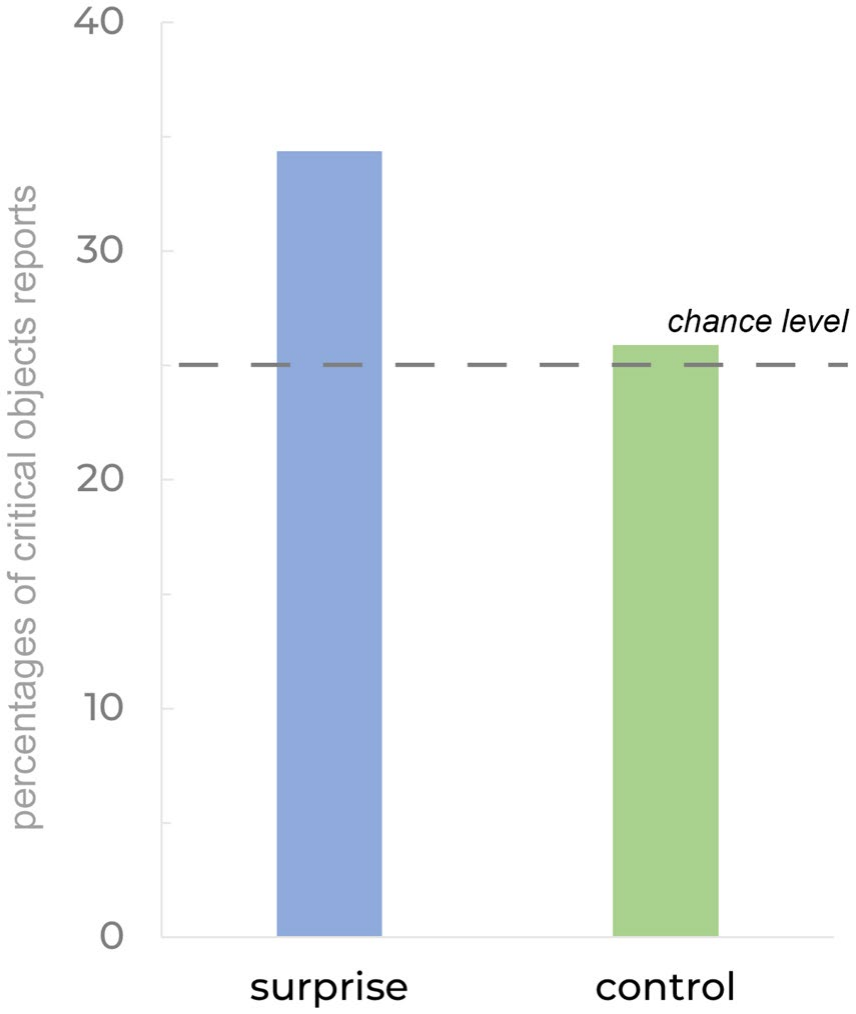
Percentages of reports matching critical object for surprise and control trials, polled across Experiments 2A-D, reflecting the probability of the foil-matching critical object being reported. Participants reported foil-matching critical object at a higher frequency than the chance level (25%) in surprise trials, but not in control trials.

In order to test whether exposure to the critical object increased the likelihood of its selection, we transformed reports in surprise and control trials into binary variables reflecting only *whether the critical object was selected* (ignoring whether this response was accurate or not). Since all four response choices are equally represented in the pooled data, the chance level is 25%. The binomial analysis revealed that percentages of reports matching the critical object were significantly higher than the chance level in surprise trials (55 reports out of 160, giving 34.375%**,** *x* = 55, *N* = 160, *p* = 0.008, Fig. 2), but not in averaged control trials (41.4 reports out of 160, giving 25.875%**,** *x* = 41, *N* = 160, *p* = 0.85, Fig. 2). These findings imply that previous exposure to an object with its identification at the category level leads to a greater propensity to choose that item in surprise trials, presumably due to increased familiarity. Below, we separately examine the role of the critical object on each of the four possible response choices (2A-2D) with the caveat that there is low power to draw substantive conclusions on differences in patterns between the studies.

### Experiment 2A. Same exemplar in the different state

#### Localization task

The location errors were higher on surprise trials compared to pre-surprise trials (42.5 vs. 6.75%, surprise trials vs. pre-surprise trials, *χ*2[1, *N* = 40] = 13.771, *p* < 0.01, *ϕ* = 0.59). Similar to Experiment 1, performance in the localization task improved in controls trials relative to surprise trials (42.5 vs. 11.5%, surprise trials vs. control trials, *χ*2[1, *N* = 40] = 9.75, *p* < 0.01, *ϕ* = 0.49).

### Identification task

**Figure 3:**
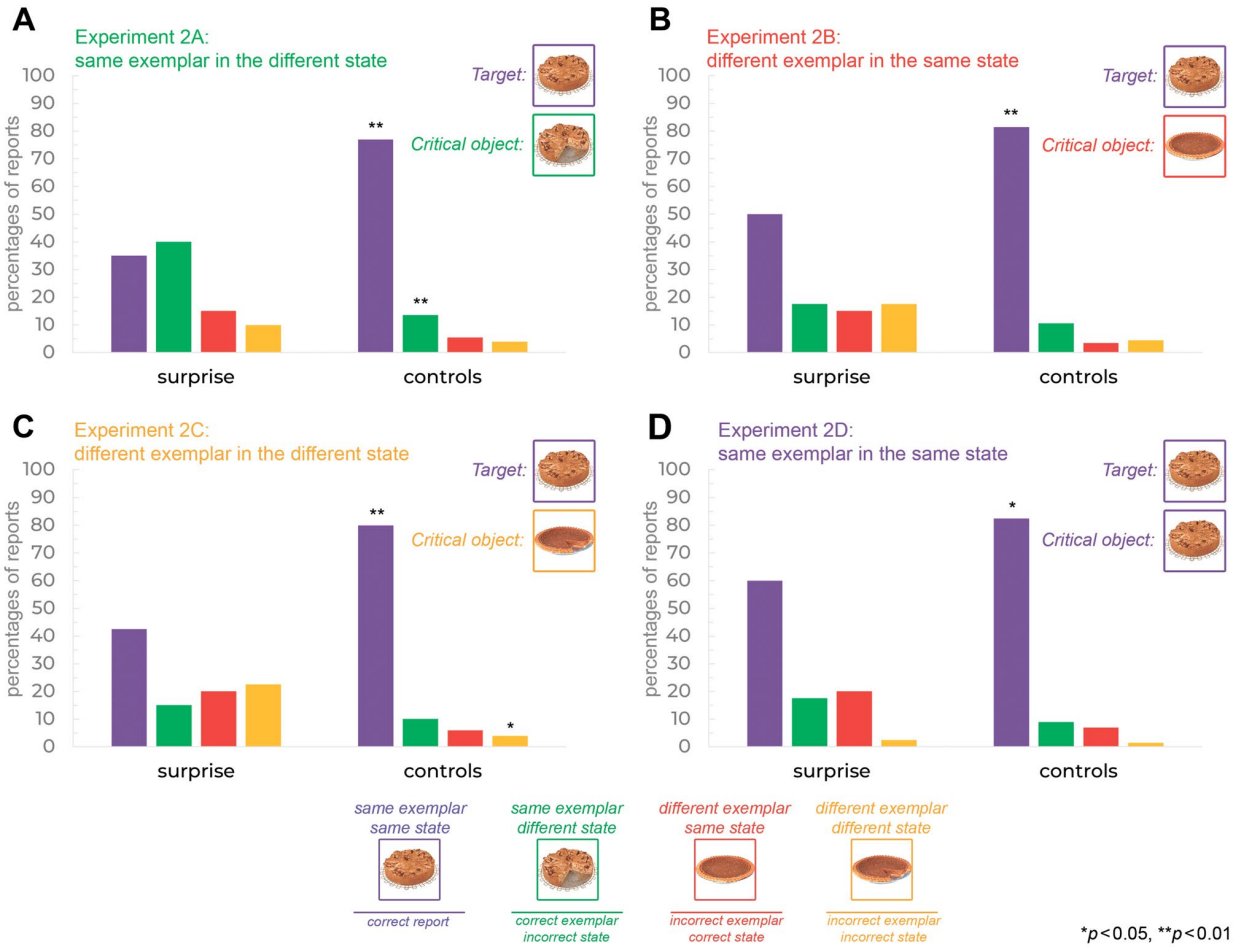
Percentage of different report types on surprise and control trials in Experiments 2A-2D. The *critical object* was a related target (food) object presented on one of the pre-surprise trials (chosen at random from the 3rd to 28th trial).

Similarly, to Experiment 1, we observed significantly fewer the *same* exemplar in the *same* state reports on surprise compared to control trials (35 vs. 77%, surprise vs. control trials, respectively *χ*2[1, *N* = 40] = 14.318, *p* = 0.0002, *ϕ* = 0.6, Fig. 3A) and also more reports of the *same* exemplar in a different state on the surprise trials compared to control trials (40 vs. 13.5%, surprise vs. control trials, respectively *χ*2[1, *N* = 40] = 7.1679, *p* = 0.007, *ϕ* = 0.423, Fig. 3A). Note that the *same* exemplar in a *different* state was the critical object, and the relatively high errors for this choice (in relation to control trials) likely reflect the influence of familiarity. There were no significant differences between surprise and control trials for a *different* exemplar in the *same* state reports (15% vs. 5.5%, surprise vs. control trials, *χ*2[1, *N* = 40] = 1.9621, *p* = 0.1613, *ϕ* = 0.22), and a different exemplar in a *different* state reports (10% vs. 4%, surprise vs. control trials, *χ*2[1, *N* = 40] = 1.106, *p* = 0.293, *ϕ* = 0.17). Overall, we replicate Experiment 1—exemplar errors were rare and not different between surprise and control trials, but state errors were higher in surprise than control trials.

### Experiment 2B. Different exemplar in the same state

#### Localization task

The location errors were higher on surprise trials compared to pre-surprise trials (35 vs. 7.6%, surprise trials vs. pre-surprise trials, *χ*2[1, *N* = 40] = 8.9708, *p* < 0.01, *ϕ* = 0.47). Performance in the localization task improved in controls trials relative to surprise trials (35% vs. 15.5%, surprise trials vs. control trials, *χ*2[1, *N* = 40] = 4.0293, *p* = 0.04472, *ϕ* = 0.32).

### Identification task

Similarly, to Experiment 2A and Experiment 1, we observed significantly fewer the *same* exemplar in the *same* state reports in surprise compared to controls trials (50 vs. 81.5%, surprise vs. control trials, respectively χ2[1, N = 40] = 8.8124, *p* = 0.003, ϕ = 0.47, Fig. 3B). In contrast to Experiment 2A and Experiment 1, here the *same* exemplar in a *different* state reports on surprise trials were not higher than on control trials (17.5% vs. 10.5%, surprise trials vs. pre-surprise trials, *χ*2[1, *N* = 40] = 0.814, *p* = 0.367, *ϕ* = 0.14, Fig. 3B). There were also no significant differences between surprise and control trials for a *different* exemplar in the *same* state reports (15 vs. 3.5%, surprise trials vs. control trials, χ2[1, N = 40] = 3.151, *p* = 0.07588, *ϕ* = 0.28). The *different* exemplar in the *same* state was the critical object so we would have expected more frequent selection of this foil in the surprise trial. While the frequency of these errors was about five times higher in surprise than in control trials, this did not reach significance. It is difficult to draw a substantive conclusion from this null, given the low power afforded by having only one surprise trial per participant.

### Experiment 2C. Different exemplar in a different state

#### Localization task

The location errors were higher on surprise trials compared to pre-surprise trials (27.5 vs. 7.3%, surprise trials vs. pre-surprise trials, *χ*2[1, *N* = 40] = 5.6551, *p* = 0.0174, *ϕ* = 0.38). Performance in the localization task improved in controls trials relative to surprise trials. This comparison almost reached the significance level (27.5 vs. 10.5%, surprise trials vs. control trials, *χ*2[1, *N* = 40] = 3.7557, *p* = 0.05263, *ϕ* = 0.31).

### Identification task

Similarly to previous experiments, we observed significantly fewer the *same* exemplar in the *same* state reports in surprise compared to control trials (42.5% vs. 80%, surprise vs. control trials, respectively *χ2*[1, N = 40] = 11.85, *p* = 0.0006, *ϕ* = 0.54, Fig. 3C). In contrast to previous Experiments, there were significantly more reports of a *different* exemplar in a *different* state on surprise than in control trials (22.5 vs. 4%, surprise trials vs. control trials, χ2[1, *N* = 40] = 5.9551, *p* = 0.01467, *ϕ* = 0.39, Fig. 3C). This likely reflects the fact that this item was the critical object and caused a higher likelihood of this item’s selection on surprise trials. The other type of reports: the *same* exemplar in a *different* state (15 vs. 10%, surprise trials vs. control trials, *χ*2[1, *N* = 40] = 0.45714, *p* = 0.499, *ϕ* = 0.11), a *different* exemplar in the *same* state (20 vs. 6%, surprise trials vs. control trials, χ2[1, *N* = 40] = 3.466, *p* = 0.06264, *ϕ* = 0.3) did not differ significantly between surprise and control trials.

### Experiment 2D. Same exemplar in the same state

#### Localization task

The location errors were higher on surprise trials compared to pre-surprise trials (42.5 vs. 6%, surprise trials vs. pre-surprise trials, *χ*2[1, *N* = 40] = 14.505, *p* < 0.01, *ϕ* = 0.6). Performance in the localization task improved in controls trials relative to surprise trials (42.5 vs. 4.5%, surprise trials vs. control trials, *χ*2[1, *N* = 40] = 16.065, *p* < 0.01, *ϕ* = 0.63).

### Identification task

In contrast to experiments reported above, here, all type of reports were not significantly different between surprise and control trials: the *same* exemplar in a *different* state, (17.5% vs. 9%, surprise trials vs. control trials, *χ*2[1, *N* = 40] = 1.2571, *p* = 0.2622, *ϕ* = 0.17, Fig. 3D), a *different* exemplar in the *same* state, (20 vs. 7%, surprise trials vs. control trials, *χ*2[1, *N* = 40] = 2.8945, *p* = 0.089, *ϕ* = 0.27), a *different* exemplar in a *different* state, (2.5 vs. 1.5%, surprise trials vs. control trials, χ2[1, N = 40] = 0.10204,* p* = 0.7494, *ϕ* = 0.05). The percentages of the *same* exemplar in the *same* state reports were lower in surprise trials compared to control trials (60% vs. 82.5%, surprise trials vs. control trials, χ2[1, N = 40] = 4.9428,* p* = 0.0262, *ϕ* = 0.35). We interpret this as reflecting the relatively low proportion of errors overall, presumably due to the fact that the critical object was the correct response, which induced participants to select the correct response more often.

## General discussion

It is tempting to think that information we attend to will be in our memory a few seconds later. However, the AA effect shows that people cannot report a particular stimulus attribute immediately after processing and focusing on this attribute^8,9^. This effect is believed to reflect a lack of working memory consolidation. Because the attended attribute is not expected to be required later, it is accessed without being consolidated. However, not all objects appear to be equal regarding AA. Unique meaningful objects have been suggested to be immune to AA (^17^, but see^32^). However, it is unclear why meaningful objects might be immune to AA. One possibility is that meaningful objects are more effortlessly encoded into visual working memory, even when identity information is not expected to be relevant later. Alternatively, it could be that meaningful objects avoid the *appearance* of AA because performance in the task can be assisted by LTM even if the attribute was not encoded in working memory. We believe that previous research is more consistent with the latter possibility. Specifically, previous studies show that AA is reduced when the meaningful objects are unique (do not repeat across trials), but AA appears when the same objects are repeated across trials^17,18^. Repeating stimuli has only a marginal^33^ or sometimes modest^34^ effect on working memory performance but reduces possible contribution from LTM, since one cannot tell whether a sense of familiarity is from the current or recent trial^35^.

To further explore AA for meaningful objects, we conducted two sets of studies. In Experiment 1, we explored the role of probe response distinctiveness on response choices in an AA paradigm. Previous studies on meaningful stimuli have used semantically and visually distinctive objects at surprise test. While performance is good under such circumstances, this leaves open the question of whether AA may be observed if the test options are less distinct and more sensitive. To explore this, we altered the AA paradigm into a four-alternative forced choice task where we crossed differences in both exemplar (different exemplars of the object from the same category, e.g., a different exemplar of pie: apple or pumpkin pie) and state (the same exemplars in different states, e.g., pies could be full or cut). We found an AA for state judgements—participants made more errors in state judgements in surprise than in control trials. However, exemplar errors were quite low and did not differ between surprise and control trials. We found that an AA for meaningful objects can occur, even under conditions where other studies have failed to observe AA, but that it requires a more sensitive test, such as state discrimination.

The results of Experiment 1 also help to elucidate which properties of memory representations for meaningful stimuli survive AA. Previous studies have shown that AA for meaningful objects is absent when the surprise question is a sub-categorical distinction (e.g., dresser vs. couch)^17^, showing that basic conceptual information can survive a surprise test. The present work goes further by suggesting that exemplar-level information (e.g., spinach vs. kale) was also not impacted during surprise trials. Therefore, whatever memory representation persists under AA, it appears to contain relatively intact exemplar, but not state information. If the representation that survived surprise was based on active memory, the most plausible scenario would be a lack of AA for both exemplar and state information. Active memory should protect state information from being lost (as in control trials). Thus, surviving representation is likely a form of LTM that is more than gist but lacks, or the memory signal is too weak for, specific details such as a state.

To test the role of LTM in the AA paradigm more directly, we artificially manipulated familiarity in Experiments 2A-D. Specifically, one of the four response choices of the surprise trial was a target in one of the pre-surprise trials (critical object). As predicted, despite not being aware of the repetition, participants selected the critical object at a higher frequency than the chance level. Critically, such a single repetition would not influence the active maintenance of the memory item but instead is expected to influence long-term memory by providing a stronger familiarity signal. By showing that familiarity can influence response choices in the AA paradigm, we demonstrate that performance is not dependent on an active memory but on LTM. Altogether, our findings give strong support to the idea that an absence of AA for meaningful stimuli could be due to the contribution of LTM and not that the attributes of meaningful objects are better encoded into an active working memory. Our findings are in line with previous work showing that meaningful stimuli can effectively access LTM and leave behind lingering memory traces even when the task does not require memorization^36–39^. For example, LTM representations can be incidentally created for objects encountered during visual search tasks: such as targets, target-related distractors, salient distractors, or even non-targets^37,40^.

The current findings are consistent with a distinction between working memory and LTM in terms of top-down control over the contents of memory. There is considerable evidence, even outside the AA paradigm, that consolidation of information in WM depends on task requirements^41,42^. On the contrary, LTM is less amenable to task requirements. It can automatically record meaningful information (including some features but not necessarily specific details), which can persist and potentially have a harmful^34^ or beneficial^43^ impact on memory.

Our findings are also suggestive of potential differences between the exemplar and state information in the strength of representation in LTM. We found significant errors on surprise trial (vs. control) for state judgments but not for exemplar judgments. This finding may suggest that LTM storage of specific details of objects, such as state, is more dependent on intentional memorization than exemplars. One possibility is that state information is more susceptible to forgetting than exemplar information and that this information was lost due to processing requirements on the surprise trial. This explanation would align with accounts positing hierarchical organization of memory representations, with exemplar information being on a higher level than state information. The hierarchical format of memory has been advocated by previous studies^30,44–47^. For example, recent LTM studies^44^ argue whether exemplar and state are retained in LTM in a dependent or independent manner, but both agreed that exemplars could be remembered without the state property, but not vice versa, assuming the hierarchical organization of the memory.

Another potential explanation, drawing on a signal detection framework^48–50^ is that the disparity between exemplars and state errors is due to differences in memory strength required to make these judgments. The state foils might be more similar to each other as they share more detail. Thus, the LTM signals that remain under AA may be too weak for accurate state judgments, but may be sufficient to discriminate between exemplar foils.

However, whether and how the similarity between exemplar versus state pairs influences the recognition test is unclear. Previous work showed that exemplar versus state discriminations have the same difficulty level^1^. Moreover, even when some evidence is provided for state pairs being more similar than exemplars, this does not necessarily explain performance in a memory test^30^. On the other hand, previous work by Draschkow et al.^51^ provided evidence that incidental LTM is better for exemplars than states. Moreover, they showed that observers subjectively rated different states of the same object as being more similar than two objects representing different exemplars. Hence, we cannot exclude the possibility that poorer performance for the state than exemplar property at least partially stemmed from state pairs being visually more similar than exemplar pairs and thus leading to stronger competition between familiarity signals, which would require stronger memory.

To summarize, the current work posits that meaningful objects are not immune to AA because meaningfulness improves working memory consolidation but that they only appear immune to AA due to an effective contribution of LTM. We have argued that the properties of the surviving memory signals are like that expected by familiarity signals in LTM—they contain exemplar information but lack, or they are not strong enough for specific details. Finally, we have provided direct evidence that familiarity can drive responses in the AA paradigm (repeating a probe choice once in a prior trial was sufficient to produce a large bias toward that response). Thus, we argue that the specialness of meaningful objects is not that they are immune to AA, but that they can bypass AA by relying on familiarity.

## Data Availability

All data and stimuli are publicly available at https://osf.io/q6g7j/.
